# Diagnosis of suid herpesvirus encephalitis using next-generation sequencing: A case report

**DOI:** 10.1097/MD.0000000000047022

**Published:** 2026-01-16

**Authors:** Wenjun Shao, Qianlin Zhang, Xiaoyang Liu, Yong Yao, Jiewen Zhang

**Affiliations:** a Department of Neurology, Henan Provincial People’s Hospital, Zhengzhou University People’s Hospital, Zhengzhou, China.

**Keywords:** Aujeszky disease, pseudorabies, SuHV-1

## Abstract

**Rationale:**

The diagnosis of viral encephalitis by conventional and biochemical results of lumbar puncture of cerebrospinal fluid (CSF) is common in neurology, but the diagnosis of specific pathogens has benefited from the improvement of CSF diagnosis technology. Second-generation sequencing allows for more sensitive and accurate pathogen detection. A case of suid herpesvirus encephalitis was reported here.

**Patient concerns:**

A 35-year-old male experienced severe right head pain and intermittent right upper limb convulsions, occurring approximately 10 times daily since January 7, 2019. He was suddenly losing consciousness, with twitching limbs, eye movement, trismus, foaming at the mouth, and urinary incontinence, lasting 10 minutes/episode. On January 10, 2019, the patient was transferred to the intensive care unit because of epilepsy symptoms were not well controlled, and the highest temperature reached 39°C. The patient had a history of blood contact while handling raw pork, resulting in scratched and bleeding fingers before the illness began.

**Diagnoses:**

Diagnostic tests revealed elevated neutrophils, low lymphocytes, high erythrocyte sedimentation rate, low free triiodothyronine levels, and high creatinine kinase. Cranial magnetic resonance imaging showed abnormalities in various brain regions, mainly affecting the gray matter structures of the cortex. CSF analysis detected suid herpesvirus-1 as the infection source.

**Interventions:**

Treatment included mannitol, sodium valproate, acyclovir, foscarnet sodium, and nutritional support.

**Outcomes:**

After 1.5 months, the patient gradually regained consciousness but remained with visual impairment and paralysis.

**Lessons:**

Patients with pork contact and clinical, laboratory, and imaging examinations suggesting viral encephalitis should be considered to have suid herpesvirus encephalitis, which can be confirmed by next-generation sequencing.

## 1. Introduction

Suid herpesvirus belongs to the *Varicellovirus* genus, mainly infecting pigs.^[1]^ Cross-species infection of other mammals, such as dogs, cattle, sheep, and mice, has been reported.^[2,3]^ Cases of suid herpesvirus encephalitis have been reported to jump from livestock to humans, leading to death in many cases due to necrotizing brain lesions or aberrant immune response.^[4,5]^

In addition, the literature was reviewed to provide evidence that suid herpesvirus can cross species to infect humans.

## 2. Case presentation

A 35-year-old male complained of headache, episodic loss of consciousness, and limb convulsion for 6 days. He had a right head intermittent dull pain and right upper limb intermittent convulsions from January 7, 2019, without conscious barriers, lasting 2 to 3 minutes each time, about 10 times a day, but had a sudden loss of consciousness with limbs twitching, turning eyes, trismus, foaming at the mouth, and urinary incontinence, lasting for 10 minutes, and with an seriousness of consciousness at that night. He was immediately taken to the local hospital. Head computed tomography showed no obvious abnormality. He had 2 similar epileptic seizures after admission. His temperature was 37.7°C. A lumbar puncture was performed. The pressure was 110 mm H_2_O. The cerebrospinal fluid (CSF) was colorless and transparent. The CSF cell number was <10 × 10^6^/L, CSF protein was 0.35 g/L, CSF glucose was 4 mmol/L, and CSF chlorine was 127.4 mmol/L. Head magnetic resonance imaging (MRI) showed an abnormally high signal in the left insular, temporal, and parietal lobes.

The patient was transferred to the intensive care unit on January 10, 2019, because of epilepsy symptoms were not well controlled, and his highest temperature was 39°C. The patent was given antiviral drugs, glucocorticoids, and immunoglobulin. There was no obvious improvement, and due to repeated seizures, the patient was considered to have aspiration pneumonia. He was then transferred to the neurology intensive care unit of our hospital (Henan Provincial People’s Hospital) on January 13, 2019.

After asking about the medical history again, the patient has been engaged in the pork business since December 2018. The patient had a history of blood flow contact because his fingers had been scratched and bleeding during the processing of raw pork handling before the onset of the illness.

**Figure 1.**
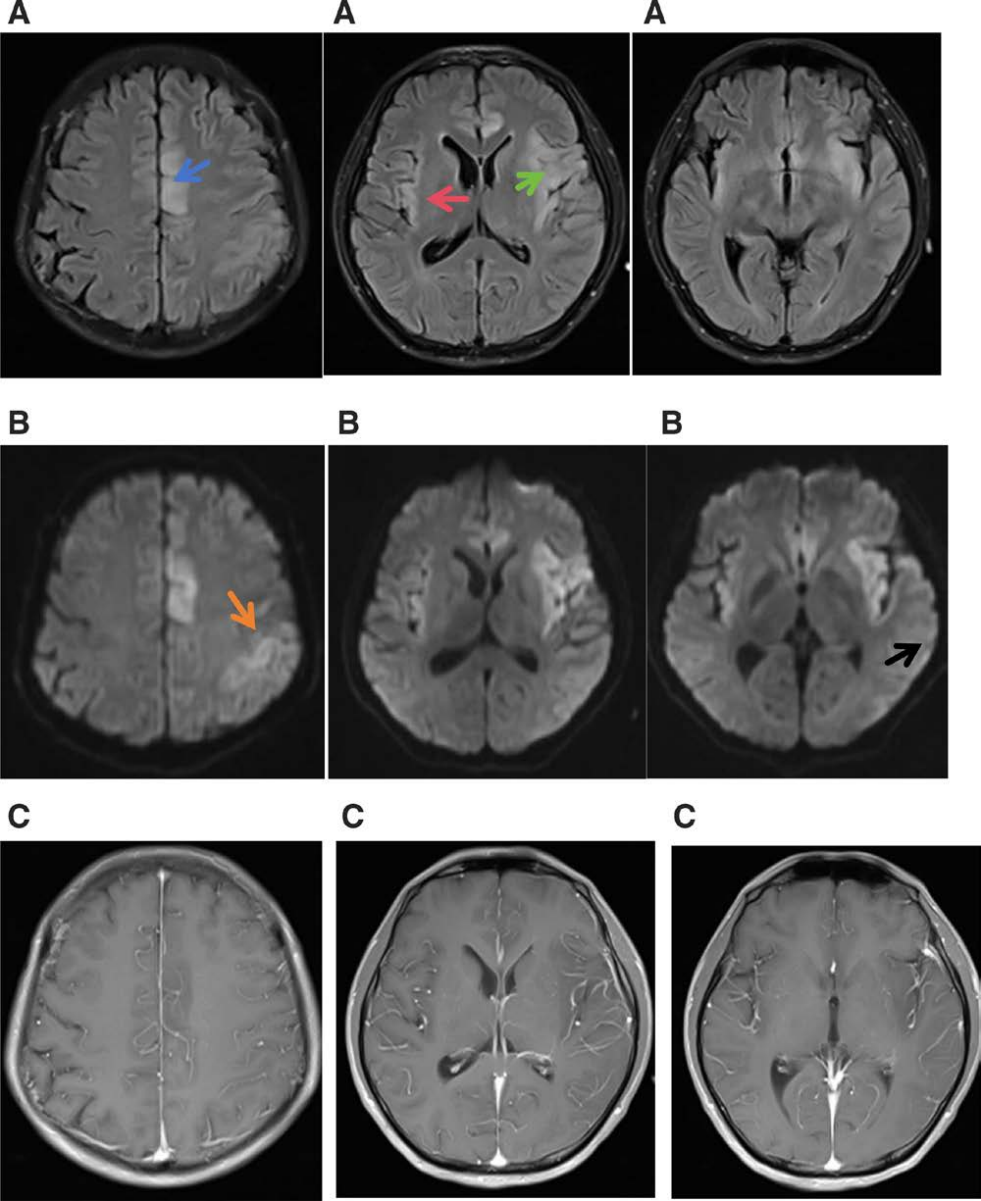
Cranial magnetic resonance imaging (MRI) showing T2 FLAIR, DWI, and enhanced T1 hyperintensity in the left frontotemporal, parietal, and occipital cortex and bilateral insula and the left cingulate gyrus. The lesion mainly involved the gray matter structure of the cortex. An enhanced scan shows meningeal enhancement. The blue arrow in (A1) indicates the FLAIR high signal in the left cingulate gyrus area. In (A2), the red arrow indicates FLAIR high signals in the right insula, while the green arrow indicates FLAIR high signals in the left insula and left frontotemporal lobe. The high DWI signal indicated by the orange arrow in (B1) is the left parietal lobe, and the high DWI signal indicated by the black arrow in (B3) is the right occipital cortex. DWI = diffusion weighted imaging, FLAIR = fluid attenuated inversion recovery, MRI = magnetic resonance imaging.

Physical examination after admission to our hospital, the patient was in a moderate coma, with endotracheal intubation and spontaneous breathing. The bilateral pupils were equally large, about 2.5 mm in diameter, and with sensitive light reflexes. The muscle tension of the limbs was normal. There was no response to pain stimulation of limbs. Tendon reflexes of both upper limbs were normal, but with hyperreflexia of both lower limbs. Pathological signs were negative on both sides, but the meningeal irritation sign was positive. Laboratory results showed neutrophils 8 × 10^9^/L (reference, 1.8–6.3 × 10^9^/L), neutrophils 96.1% (reference, 40%–75%), lymphocytes 0.21 × 10^9^/L (reference, 1.1–3.2 × 10^9^/L), erythrocyte sedimentation rate 38 m/h (reference, 0–20 mm/h), free triiodothyronine 1.93 pmol/L (reference, 3.1–6.8 pmol/L), and creatine kinase 1254 IU/L (reference, 55–170 IU/L). On January 14, 2019, a CSF examination showed white blood cells at 98 × 10^6^/L (reference, 0–8 × 10^6^/L), multinucleate cells at 14%, and monocytes at 86%. CSF biochemistry was normal. CSF tuberculosis gene Xpert showed that the *Mycobacterium tuberculosis* complex group was negative. The nucleic acid quantification of single vesicular virus type I, type II, and Epstein-Barr virus was negative. Cranial MRI (January 15, 2019) showed long T1 weighted image, long T2 weighted image, and fluid attenuated inversion recovery, and diffusion weighted imaging hyperintensity in the left frontotemporal, parietal, and occipital cortex, bilateral insula, and left cingulate gyrus, and the lesion mainly involved the gray matter structure of the cortex (Fig. 1). The radiologist suggested abnormal signals of the left frontotemporal, parietal, and occipital cortex and bilateral insular cortex; and pia-arachnoid enhanced shadow increase.

The high-throughput gene detection report of the CSF infection source of the patient indicated the only presence of suid herpesvirus-1 (SuHV-1). The differential diagnosis was made with autoimmune encephalitis, other central nervous system infections, and paraneoplastic autoimmune encephalitis. Therefore, this patient was considered for clinical diagnosis of porcine herpesvirus type I infectious viral meningitis. However, further virus isolation or validation is lacking.

The patient was given mannitol for dehydration and reducing craniocerebral pressure, sodium valproate and levetiracetam for antiepileptic, acyclovir, and foscarnet sodium for antiviral treatment, and nutritional support. About 3 days later, the patient changed from a coma to a mild coma, and after 1 week, he could open his eyes when called, but was unable to understand or communicate. Glasgow Coma Scale increased from 4 to 6 points. In addition, the patient’s pupils changed greatly, with a diameter of 4 to 5 mm, and the light reflex was dull and weakened. The patient was transferred to the rehabilitation department for further rehabilitation treatment after his condition was stable. At the 2-month follow-up, the patient was conscious but unable to communicate and had mixed aphasia. At the 3-month follow-up, the patient could communicate with his family verbally.

## 3. Discussion and conclusions

SuHV-1 is neurotropic, can effectively move into the peripheral nervous system, and establish a lifelong incubation period in the ganglion neurons.^[1]^ The virus is absorbed by sensory nerve endings, then reaches sensory nerve ganglia first by axonal retrograde transport and then the central nervous system.^[2]^ The patient reported here injured his fingers but continued to work and handled raw pork meat. The virus was probably present in the meat and reached the sensory nerve endings through the injuries.

Infection with the typical SuHV-1 in dogs showed that the infection does not lead to suppurative inflammation^[4]^ but is characterized by mononuclear cells around vascular proliferation, gliosis, degeneration of neurons, and virus inclusion body formation in astrocytes.^[4]^ Still, most neurons appear to remain unaffected, although a few show enrichment of eosinophilic cytoplasm and nuclear pyknosis.^[4]^ In the case reported here, brain histology was not assessed, but the CSF cytology and biochemistry were normal. MRI also showed lesions in specific areas of the brain (left insular, temporal, and parietal lobes).

Infection of humans with SuHV-1 is rare.^[3,5]^ In 2017, second-generation sequencing technology was applied to detect SuHV-1 in the vitreal fluid of a patient with endophthalmitis, and showed for the first time that SuHV-1 could infect humans.^[6]^ A multicenter encephalitis collaborative group demonstrated that SuHV-1 could cause human encephalitis.^[7]^ In the patient reported here, the initial microbiological examinations were negative, but high-throughput sequencing finally revealed SuHV-1 in the CSF. The patient was in a coma, the bilateral pupils were large, and the light reflex was weak. Even after regaining consciousness, the patient had vision impairments, probably related to neurological and optic nerve damage caused by SuHV-1.

In addition to the transmission route, the pathogenicity of the virus is closely related to the variation and evolution of the virus strain. SuHV-1 contains over 70 functional genes that encode proteins involved in the viral capsid, tegument, and envelope formation.^[8]^ Among these proteins, envelope components glycoprotein B and glycoprotein C induce cellular and humoral immune responses,^[9]^ which play a central role in immune induction, while gE is a major determinant of SuHV-1 toxicity in pigs,^[10]^ which determines the tendency of the central nervous system. These 3 genes are commonly used to monitor the evolution and variation of SuHV-1.^[11]^

At present, there is no available treatment guideline for SuHV-1 encephalitis treatment.^[12]^ The patient reported here received support treatments for antiviral therapy, reducing intracerebral pressure, preventing seizures, and maintaining nutritional status. Acyclovir and foscarnet are for antiviral treatment, but whether they are effective against SuHV-1 is mostly unknown. Zhou et al^[12]^ treated 2 patients with SuHV-1 infection and reviewed the literature for 18 other cases. All 20 patients received antiviral treatment after the initial diagnosis of viral encephalitis: acyclovir (n = 17), foscarnet (n = 6), ganciclovir (n = 2), and penciclovir (n = 1). Other treatments included intravenous immunoglobulin (n = 2), glucocorticoids (n = 3), and intravenous immunoglobulin and glucocorticoids (n = 9). Most patients responded poorly to acyclovir and immunotherapy; they had a poor neurological prognosis, and the mortality rate was 20%. The survivors had severe disability and dysfunction.^[12]^ The patient reported here survived but was left with visual impairments. Whether acyclovir and foscarnet helped or not is unknown.

In conclusion, patients with a history of blood contact with raw pork meat and a viral encephalitis diagnosis should be considered to have suid herpesvirus encephalitis infection, which can be confirmed by next-generation sequencing. CSF or MRI examinations alone cannot confirm SuHV-1 infection. Next-generation sequencing is thus a powerful tool to identify the exact virus and strain responsible for signs and symptoms.

## Author contributions

**Conceptualization:** Wenjun Shao.

**Data curation:** Qianlin Zhang.

**Formal analysis:** Qianlin Zhang.

**Project administration:** Xiaoyang Liu.

**Supervision:** Jiewen Zhang.

**Visualization:** Jiewen Zhang.

**Writing – original draft:** Yong Yao.

**Writing – review & editing:** Jiewen Zhang.
